# An novel effective and safe model for the diagnosis of nonalcoholic fatty liver disease in China: gene excavations, clinical validations, and mechanism elucidation

**DOI:** 10.1186/s12967-024-05315-3

**Published:** 2024-07-04

**Authors:** Jida Wang, Beitian Jia, Jing Miao, Dun Li, Yin Wang, Lu Han, Yin Yuan, Yuan Zhang, Yiyang Wang, Liying Guo, Jianwei Jia, Fang Zheng, Sizhen Lai, Kaijun Niu, Weidong Li, Yuhong Bian, Yaogang Wang

**Affiliations:** 1 School of Integrative Medicine, Tianjin University of Traditional Chinese Medicine, Tianjin, 301617 People’s Republic of China; 2 Tianjin Second People’s Hospital, Department of Integrated Traditional Chinese and Western Medicine, Tianjin, 300192 People’s Republic of China; 3 Public Health Science and Engineering College, Tianjin University of Traditional Chinese Medicine, Tianjin, 301617 China; 4 School of Public Health, Tianjin Medical University, Tianjin, 300070 China; 5 Department of General Surgery, Tianjin Medical University General Hospital, Tianjin, 300052 China

**Keywords:** Nonalcoholic fatty liver disease, Machine learning, Biomarkers

## Abstract

**Background:**

Non-alcoholic fatty liver disease (NAFLD) is one of the most common chronic liver diseases. NAFLD leads to liver fibrosis and hepatocellular carcinoma, and it also has systemic effects associated with metabolic diseases, cardiovascular diseases, chronic kidney disease, and malignant tumors. Therefore, it is important to diagnose NAFLD early to prevent these adverse effects.

**Methods:**

The GSE89632 dataset was downloaded from the Gene Expression Omnibus database, and then the optimal genes were screened from the data cohort using lasso and Support Vector Machine Recursive Feature Elimination (SVM-RFE). The ROC values of the optimal genes for the diagnosis of NAFLD were calculated. The relationship between optimal genes and immune cells was determined using the DECONVOLUTION algorithm CIBERSORT. Finally, the specificity and sensitivity of the diagnostic genes were verified by detecting the expression of the diagnostic genes in blood samples from 320 NAFLD patients and liver samples from 12 mice.

**Results:**

Through machine learning we identified *FOSB*, *GPAT3, RGCC* and *RNF43* were the key diagnostic genes for NAFLD, and they were further demonstrated by a receiver operating characteristic curve analysis. We found that the combined diagnosis of the four genes identified NAFLD samples well from normal samples (AUC = 0.997). *FOSB*, *GPAT3, RGCC* and *RNF43* were strongly associated with immune cell infiltration. We also experimentally examined the expression of these genes in NAFLD patients and NAFLD mice, and the results showed that these genes are highly specific and sensitive.

**Conclusions:**

Data from both clinical and animal studies demonstrate the high sensitivity, specificity and safety of FOSB, GPAT3, RGCC and RNF43 for the diagnosis of NAFLD. The relationship between diagnostic key genes and immune cell infiltration may help to understand the development of NAFLD. The study was reviewed and approved by Ethics Committee of Tianjin Second People’s Hospital in 2021 (ChiCTR1900024415).

**Supplementary Information:**

The online version contains supplementary material available at 10.1186/s12967-024-05315-3.

## Introduction

Non-alcoholic fatty liver disease (NAFLD) is one of the most common chronic liver diseases, that affects one in four adults worldwide [1]. The current global prevalence of NAFLD is about 25.2% and is expected to reach 33.5% by 2030 [2]. At the beginning of the 21st century, the prevalence of NAFLD in China was estimated to be 23.8% (95% CI: 16.4-31.2%). In 2018, the NAFLD prevalence reached 32.9% (95% CI: 28.9-36.8%). By 2030, the total number of NAFLD patients in China is expected to increase up to 314.58 million and is expected to reach 50.8%, making it the fastest-growing country in the world in terms of NAFLD prevalence [3].

NAFLD is a clinicopathological syndrome characterized by diffuse bullae of fat in hepatocytes [4]. The inflammatory subtype of NAFLD is nonalcoholic steatohepatitis (NASH), which can progress to cirrhosis and even hepatocellular carcinoma [5]. NAFLD is a risk factor for chronic kidney disease [6], cardiovascular disease [7], and sleep apnea [8]. In addition to that, NAFLD may remain silent for several years before the manifestation of its representative symptoms and structural changes become visible in a radiography [9]. Therefore, early diagnosis of NAFLD is essential to delay the progression of the disease and for the treatment [10].

Unfortunately, there is still a lack of such early diagnostic tools in clinical practice [11]. At present, liver biopsy remains the gold standard for NAFLD diagnosis, despite the limitations regarding sampling variability, invasivity, and high cost. Early detection of hepatic steatosis is the first step in the diagnosis of NAFLD [12], and noninvasive assessment models such as the fatty liver index and the hepatic steatosis index are the initial strategies suitable for use in primary care hospitals as a screen for hepatic steatosis [13]; in terms of imaging, ultrasound, although widely available, has a low sensitivity for mild hepatic steatosis, and therefore is not recommended as a tool for screening for hepatic steatosis in the AASLD guidelines [14, 15]. Controlled attenuation parameters allow for immediate semiquantitative assessment and are gradually becoming more widely used, whereas the proton density fat fraction of MRI is the most accurate, reproducible, and precise means of quantifying hepatic steatosis, but is now used only for clinical research due to cost and patient acceptance [16]. Therefore, the development of new diagnostic biomarkers that can reflect the entity of liver injury is critical for early diagnosis and treatment of NAFLD [17, 18].

With the development of high-throughput gene microarray technology, researchers can analyze the gene and mRNA differences among samples derived from patients with a variety of diseases and from healthy humans [19]. Many specific genes involved in the progression of NAFLD have been already identified. For example, the expression level of fibroblast growth factor 21 (FGF21) in the liver of NAFLD patients is considerably lower than that of healthy individuals [20]. In a mouse model, FGF21 was associated with a decrease in the progression of NAFLD [21]. Moreover, the interference of the FGF21–mechanistic target of rapamycin complex 1 axis reduces chronic NAFLD via stimulation of FGF21 kinase, suggesting that the FGF21 pathway may be a potential therapeutic target for NAFLD [22]. Additionally, *Xie et al.*. found downregulated the expression of FK506-binding protein 38 (FKBP38) in the livers of NAFLD patients when compared with healthy livers. Furthermore [23], FKBP38 overexpression enhances the mitochondrial fatty acid oxidation defense and promotes adipose oxidation in fat cells through the mTOR/SREBPs signaling pathway [24]. These findings suggest an important role for some functional genes in NAFLD progression. However, whether these functional genes can be used for the diagnosis of NAFLD remains unclear.

In the present study, we aimed to identify novel NAFLD diagnostic genes by using bioinformatics and machine learning. A Gene Expression Omnibus (GEO) dataset (GSE89632) was used to screen differentially expressed genes (DEGs) between NAFLD and healthy specimens. Next, machine learning was used to analyze the diagnostic value of these genes in NAFLD. The expression of these genes was further examined in NAFLD animal model mice liver and clinical patients blood. We also examined the expression of these genes in hepatitis B, hepatitis C, and autoimmune hepatitis (AID) patients blood to determine their specificity.

## Materials and methods

### Microarray data

The microarray data used to establish the diagnostic model for NAFLD were the GSE89632 mRNA expression profile data (including blood samples from 19 NAFLD patients and 24 normal controls), was downloaded from GEO (https://www.ncbi.nlm.nih.gov/geo/) [25]. Screening criteria: the period of data collection was before January 2023. The dataset intelligently contained healthy people and patients with NAFLD (excluding patients with alcoholic fatty liver disease, hepatitis, liver fibrosis, and hepatocellular carcinoma). The patients with fatty liver disease had not been subjected to pharmacological interventions as at the time of data collection.

### Identification of DEGs

DEGs between NAFLD and normal samples were identified using R software with the Limma package. The thresholds for DEGs were set as |Log_2_FC|>1 and false discovery rate (FDR) < 0.05.

### Functional enrichment analyses

Gene Ontology (GO) and Kyoto Encyclopedia of Genes and Genomes (KEGG) pathway analysis were performed using “clusterProfiler” in the R software to complete the analysis of patients in the high-risk and low-risk groups. GO terms and KEGG pathways with *P* < 0.05 were considered statistically significant. Disease Ontology enrichment analysis was performed using the “clusterProfiler” and “DOSE” packages in the R software.

### Identification of candidate diagnostic markers

Two machine learning algorithms, lasso and support vector machine recursive feature elimination (SVM-RFE), were used to predict the NAFLD status using possible diagnostic factors. The lasso regression algorithm was performed to identify genes significantly associated with the discriminative power of NAFLD and healthy specimens using R software with the “GlMNet” package. Support vector machine (SVM) is a monitored machine learning algorithm that is widely used for both classification and regression analyses. Moreover, the recursive feature elimination (RFE) algorithm is used to avoid overfitting and thus screen the optimal genes from the metadata queue. Therefore, to identify the gene set with the strongest recognition ability, SVM-RFE was used to screen suitable features [26].

### CIBERSORT analysis

The deconvolution algorithm CIBERSORT (http://cibersort.stanford.edu/) was used to analyze the cell composition of complex tissue samples according to the gene expression profile of each cell type. With the help of the CIBERSORT algorithm, we identified the immune responses of 22 immune cell types and determined their relationships with the mRNA levels of key genes in normal and NAFLD samples. The purpose of this study was mainly to determine the link between these immune cells.

### Clinical specimens

Blood samples were collected from patients with NAFLD (*n* = 320, including 160 females and 160 males, with the age of 21–59 years), Hepatitis B(*n* = 289, including 145 females and 144 males, with the age of 21–59 years), Hepatitis C (*n* = 292, including 152 females and 140 males, with the age of 21–59 years) or AID (*n* = 289 per group, including 156 females and 133 males, with the age of 21–59 years) who were diagnosed according to the criteria released by the Chinese Society of Hepatology Medical Association (Table 1). Patients with hepatitis B and hepatitis C were simply infected with the virus and are not in the active phase, but have the same AST and ALT abnormalities as patients with NAFLD. Healthy blood samples were obtained from patients without any disease (*n* = 300, including 150 females and 150 males, with the age of 21–59 years). Informed consent was obtained from all cases for tissue sample use. The present work gained approval from Ethics Committee of Tianjin Second People’s Hospital.

**Table 1:** Patient demographics and biochemical index information

Classification	F/ M	AST	ALT	TG	TC	LDL-c	virus	Brightness mode
Control	150/150	25.83 ± 5.87	18.81 ± 4.66	1.62 ± 0.21	3.89 ± 0.54	2.64 ± 0.41	**-**	negative
NAFLD	160/160	92.81 ± 9.3**	136.5 ± 60.7*	2.56 ± 0.52*	6.94 ± 0.83*	4.03 ± 0.19*	-	positive
Hepatitis B	145/144	50.5 ± 8.18*	33 ± 14.3**	1.42 ± 0.8	3.79 ± 0.28	2.87 ± 1.06	+	negative
Hepatitis C	152/140	52.83 ± 17.6*	67.66 ± 2.7*	1.89 ± 0.56	4.01 ± 1.48	3.05 ± 0.84	+	negative
ALD	156/133	48.5 ± 2.18*	46.5 ± 21.7*	2.87 ± 0.42*	5.23 ± 0.76*	3.98 ± 0.77**	-	positive

Compared with the Control, NAFLD, MG Hepatitis B, Hepatitis C and ALD *P < 0.05, **P < 0.01

### Animal specimens

Twelve 8-week-old specific-pathogen-free male C57BL/6 mice weighing about 20 g were maintained under a 12-h light/dark cycle with a temperature of 22 ± 2 °C and a humidity of 50 ± 5% with free access to food and water. All animal procedures were approved by the Institutional Anima·l Care and Use Committee of the Tianjin University of Chinese Medicine. The mice were adaptively fed for seven days before the experiments and were then randomly divided into two groups: normal (fed with standard chow for 12 weeks) and NAFLD model (fed with a NAFLD/NASH high-fat-rich diet and drinking water containing fructose for 12 weeks) groups. After 12 weeks, the mice were sacrificed, and their liver samples were collected.

### Real-time polymerase chain reaction (RT-PCR) assay

Total RNA was extracted from the liver samples by TRIzol (Invitrogen), according to the manufacturer’s protocol. cDNA was first synthesized using the extracted RNA with random primers and a reverse transcription kit (Takara, China). The reverse transcription process was performed as follows: 37 °C for 15 min followed by 5s at 85 °C. Then qRT-PCR was carried out using a Power SYBR Green kit (Takara, China) on an ABI 7500 instrument. The relative mRNA level was calculated using the 2^−ΔΔCt^ method. Glyceraldehyde-3-phosphate dehydrogenase (*GAPDH*) was used as an internal control for data normalization. The primers for the genes encoding an AP-1 transcription factor subunit (*FOSB*), glycerol-3-phosphate acyltransferase 3 (*GPAT3*), regulator of the cell cycle (*RGCC*), and ring finger protein 43 (*RNF43*) as well as *GAPDH* are shown in Table 2.

**Table 2:** Primer sequences to measure mRNA levels using quantitative RT-PCR

Gene (Mus musculus)	Primer	Sequence (5′–3′)
*FOSB* (mouse)	forward	CCTCCGCCGAGTCTCAGTA
	reverse	CCTGGCATGTCATAAGGGTCA
*GPAT3* (mouse)	forward	CGGATTATCCCTGGGTATCTCG
	reverse	CGAAGTCCCTTCCTCGAAGAC
*RGCC* (mouse)	forward	GAGCGCCACTTCCACTATGAG
	reverse	GGAGAGGAGTTGGTTGGAGAA
*RNF43* (mouse)	forward	CACGAGTTTCATCGAACGTGT
	reverse	CTGGCGAATGAGGTGGAGT
*GAPDH* (mouse)	forward	AGGTCGGTGTGAACGGATTTG
	reverse	GGGGTCGTTGATGGCAACA
*FOSB* (human)	forward	GCTGCAAGATCCCCTACGAAG
	reverse	ACGAAGAAGTGTACGAAGGGTT
*GPAT3* (human)	forward	CGCTGGTTCTCGGCTTCAT
	reverse	TGGCCCACTCTAAAGTTTTCAC
*RGCC* (human)	forward	CGCCACTTCCACTACGAGG
	reverse	CAGCAATGAAGGCTTCTAGCTC
*RNF43* (human)	forward	CATCAGCATCGTCAAGCTGGA
	reverse	TTACCCCAGATCAACACCACT
*GAPDH* (human)	forward	GGAGCGAGATCCCTCCAAAAT
	reverse	GGCTGTTGTCATACTTCTCATGG

### Statistical analysis

The Student’s *t* test was used to compare the gene expression between NAFLD tissues and adjacent tissues. To test the classification effect of key genes on NAFLD and healthy samples, the “pROC” package in R software was used to calculate the receiver operating characteristic (ROC) curve and the area under the curve (AUC). Statistical analyses were performed using R software (Version 3.5.3) and Prism (GraphPad Prism, USA). A *P*-value of < 0.05 was considered statistically significant.

## Results

### Identification of DEGs in NAFLD

**Fig. 1:**
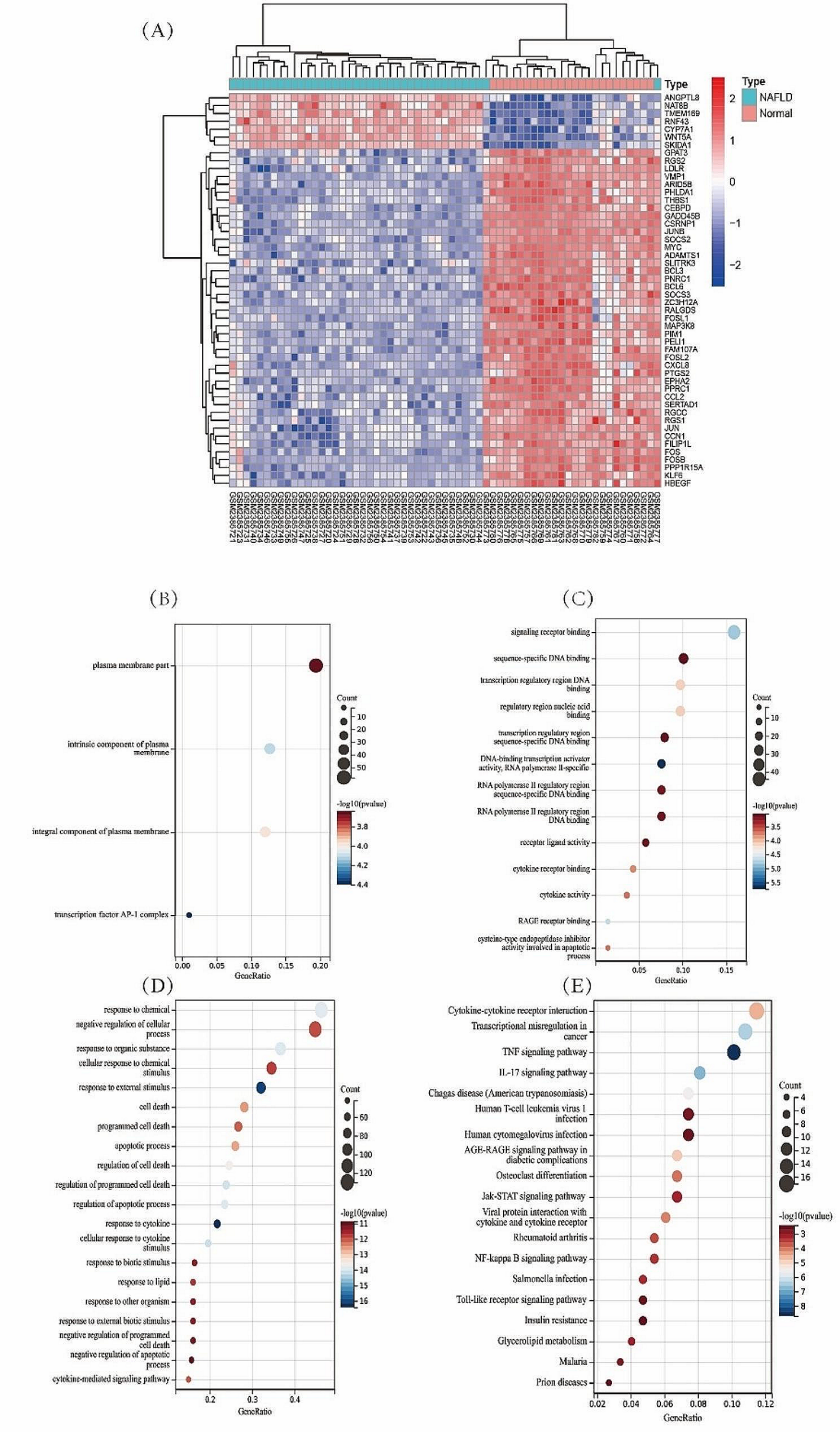
**A**: DEGs between NAFLD and healthy specimens. GO analysis (**B**: cellular components, **C**: molecular functions, **D**: biological processes) and KEGG analysis (**E**) of 334 DEGs via the ClusterProfile package of R software

Firstly, we find the differentially up-regulated or down-regulated genes in the NAFLD samples in the dataset. The data of 19 NAFLD cases and 24 controls from the GEO dataset (GSE89632) were included for analysis. After the batch effect was removed, the DEGs of the metadata were analyzed by R software with the Limma package. We identified 334 DEGs, including 223 upregulated genes and 111 downregulated genes (Fig. 1A).

### Functional enrichment analyses

Next, GO and KEGG analyses were performed for these 334 DEGs using R software with the Cluster Profile package. The results showed that the 334 DEGs mainly participated in the response to chemicals, negative regulation of cellular processes, response to organic substances, cellular response to chemical stimuli, response to external stimuli, cell death, programmed cell death, signaling receptor binding, sequence-specific DNA binding, transcription regulatory region DNA binding, the plasma membrane part, the intrinsic component of the plasma membrane, and the integral component of the plasma membrane (Fig. 1B–D). Meanwhile, KEGG analysis showed that the following pathways were significantly enriched: cytokine–cytokine receptor interactions, the interleukin (IL)-17 signaling pathway, the tumor necrosis factor (TNF) signaling pathway, and transcriptional dysregulation in cancer (Fig. 1E).

### Identification of diagnostic marker candidates

**Fig. 2:**
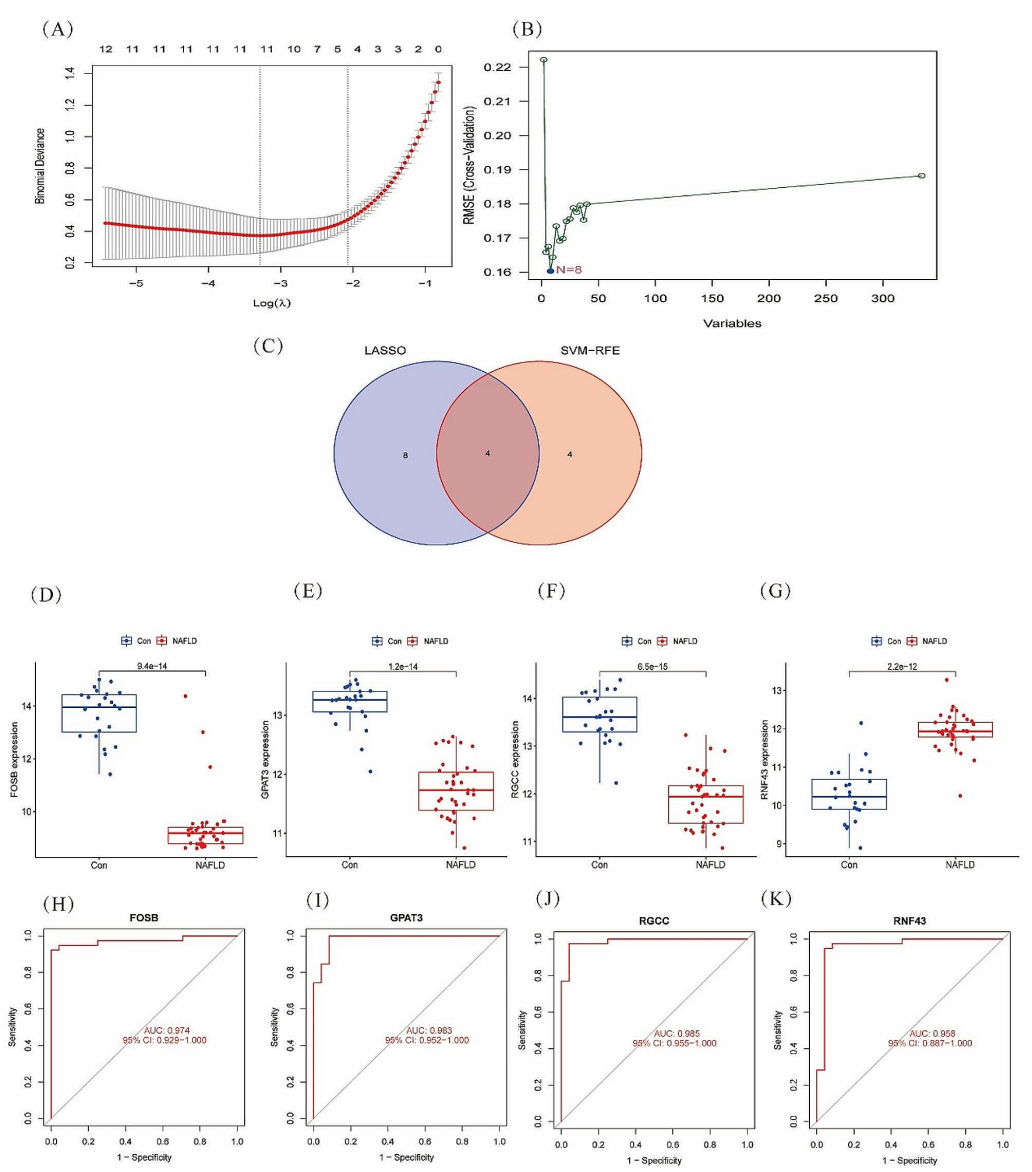
Identification of the gene candidates for NAFLD diagnosis: (**A**) tuning feature selection using the lasso model; (**B**) the screening of gene candidates by the SVM-RFE algorithm; (**C**) The Venn diagram presenting for gene candidates shared by lasso and SVM-RFE. Expression of *FOSB*(**D**), *GPAT3*(**E**), *RGCC*(**F**), and *RNF43*(**G**) in the common human and NAFLD in the dataset. Expression of *FOSB*(**H**), *GPAT3*(**I**), *RGCC*(**G**), and *RNF43*(**K**) in the common human and NAFLD in the dataset

Two different algorithms were used to identify potential biomarkers. SVM-RFE algorithm and lasso regression algorithm are the core independent variables in screening differential genes, which can reflect the characteristics of the disease. These DEGs were analyzed by the lasso regression algorithm, which determined 12 genes as diagnostic markers for NAFLD (Fig. 2A). Through the SVM-RFE algorithm (Fig. 2B), a subset of eight genes was determined using the difference analysis object. Finally, four overlapping genes (*FOSB*, *GPAT3*, *RNF43*, and *RGCC*) were identified with the two algorithms (Fig. 2C). These four genes (*FOSB*, *GPAT3*, *RNF43*, and *RGCC*) may be the key genes involved in the progression of NAFLD.

### The expression and diagnostic value of FOSB, GPAT3, RNF43, and RGCC in NAFLD

Compared with the healthy samples, the expression levels of *FOSB*, *GPAT3*, and *RGCC* were significantly downregulated in the NAFLD samples (Fig. 2D, E, F); whereas the expression level of *RNF43* was significantly upregulated in the NAFLD samples (Fig. 2G). Next, ROC curve analysis was performed to evaluate the diagnostic value of *FOSB*, *GPAT3*, *RGCC*, and *RNF43*. We found that the AUCs of *FOSB* (Fig. 2H), *GPAT3* (Fig. 2I), *RGCC* (Fig. 2J), and *RNF43* (Fig. 2K) were 0.974, 0.983, 0.985, and 0.958, respectively, suggesting that all four genes showed a strong ability to differentiate NAFLD samples from normal samples.

**Fig. 3:**
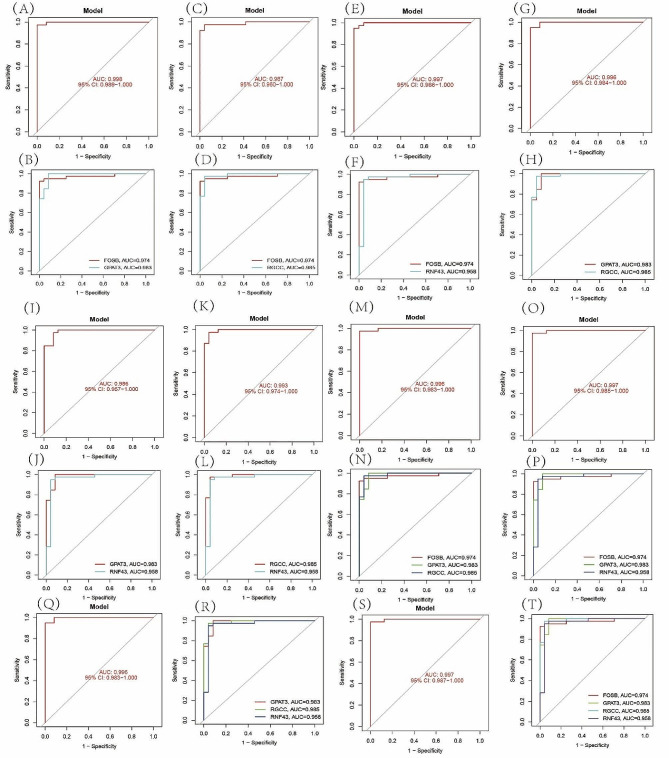
The ROCs of two, three and four genes. (**A** - **L**) The ROCs of combined two genes. (**N** - **R**) The ROCs of combined three genes. (**S** - **T**) The ROCs of combined four genes

Then, ROC curve was used to analyze the diagnostic value of combined two, three and four genes for NAFLD. We found that two, three and four genes exhibited a strong ability in screening NAFLD samples from normal samples (Fig. 3A, B AUC = 0.998; C, D AUC = 0.997; E, F AUC = 0.997; G, H AUC = AUC = 0.996; I, J AUC = 0.986; K, L AUC = AUC = 0.993; M, N AUC = 0.996; O, P AUC = AUC = 0.997; Q, R AUC = 0.996; S, T AUC = 0.997;) Each gene alone was used to diagnose NAFLD at a very high level, and the combination of all four genes had the highest ROC value for the diagnosis of NAFLD.

### FOSB, GPAT3, RGCC, and RNF43 are associated with the infiltration levels of immune cells

**Fig. 4:**
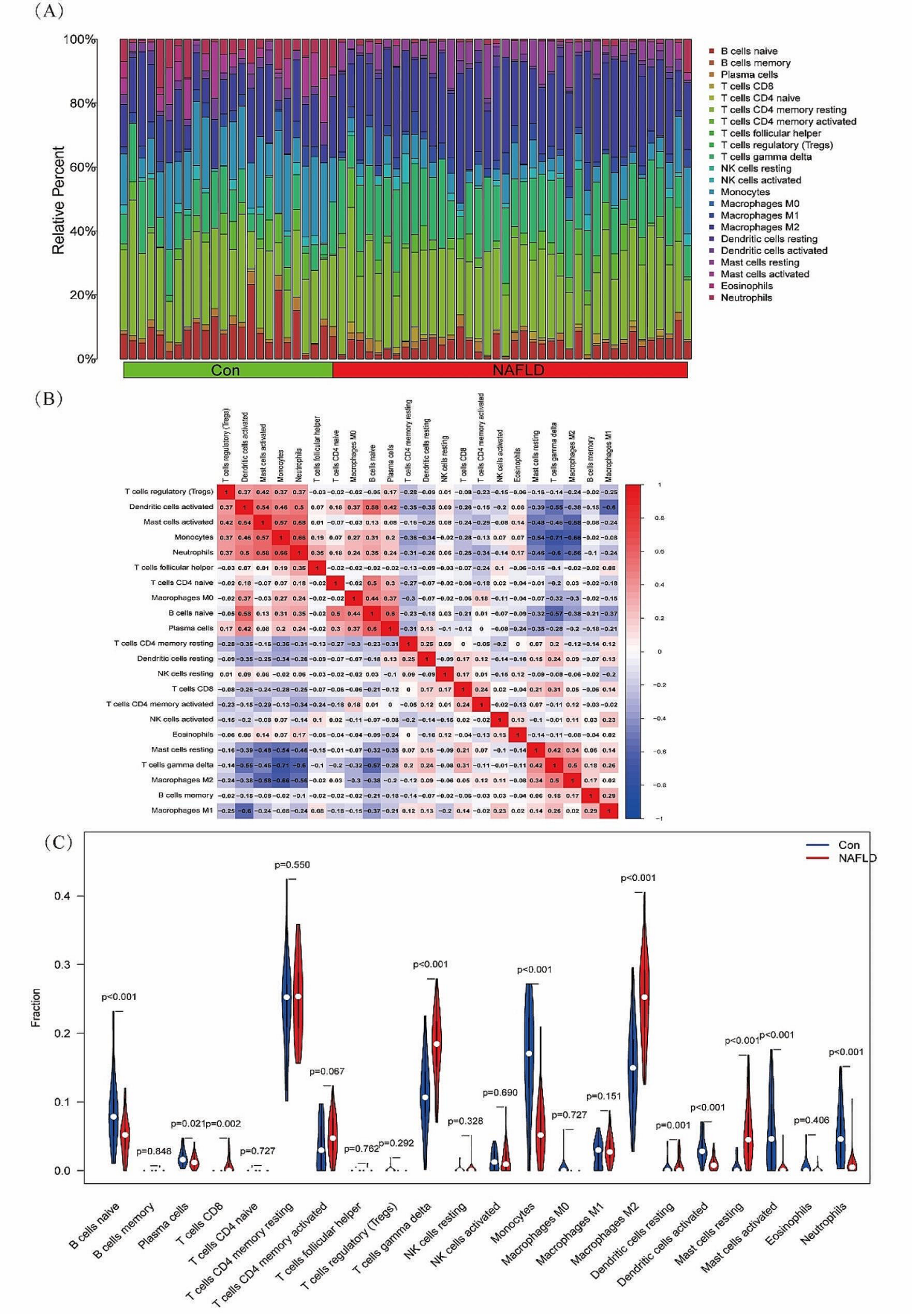
Relationship between four genes (FOSB, GPAT3, RGCC and RNF43) and infiltration levels of immune cells. (**A**, **B**) The percentage of the 22 immunocytes identified using CIBERSORT. (**C**) The differences in the composition of immunocytes between healthy and NAFLD samples

Immune cell infiltration in the tumor microenvironment is an independent predictor of overall survival and prognosis. Therefore, we investigated the coefficients of *FOSB*, *GPAT3*, *RGCC*, and *RNF43* and the infiltration status of immune cells in NAFLD and normal samples to determine the correlation between them. The CIBERSORT method was used to analyze the characteristics of immune cells. The composition of the immune cells in NAFLD and normal specimens as well as the relationships between immune cells are presented in Fig. 4A–B. The levels of CD8^+^ T cells, resting dendritic cells were significantly different between the normal and NAFLD samples (Fig. 4C).

**Fig. 5:**
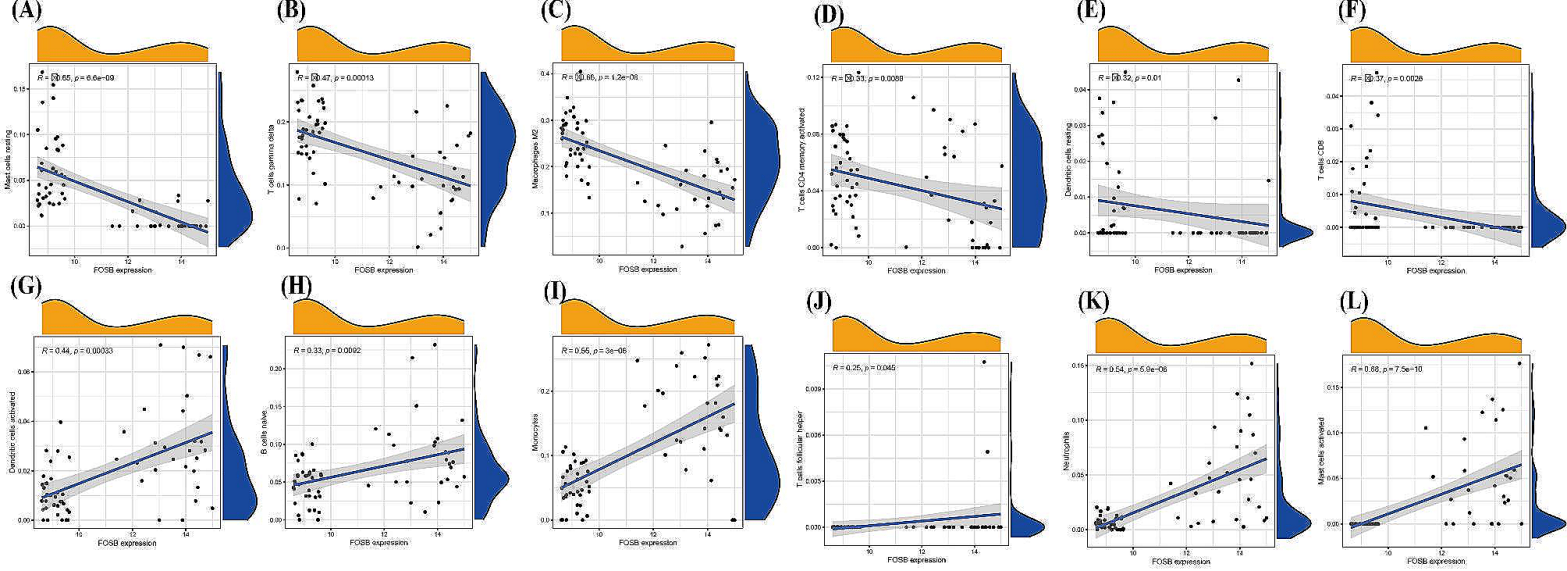
Correlation between FOSB and infiltrating immune cells in NAFLD and healthy samples. (A. Mast cells resting), (B. T cells gamma delta), (C. Macrophages M2), (D. T cells CD4 memory activated), (E. Dendritic cells resting), (F. T cells CD8), (G. Dendritic cells activated), (H. B cells naive), (I. Monocytes), (J. T cells follicular helper), (K. Neutrophils), (L. Mast cells activated)

**Fig. 6:**
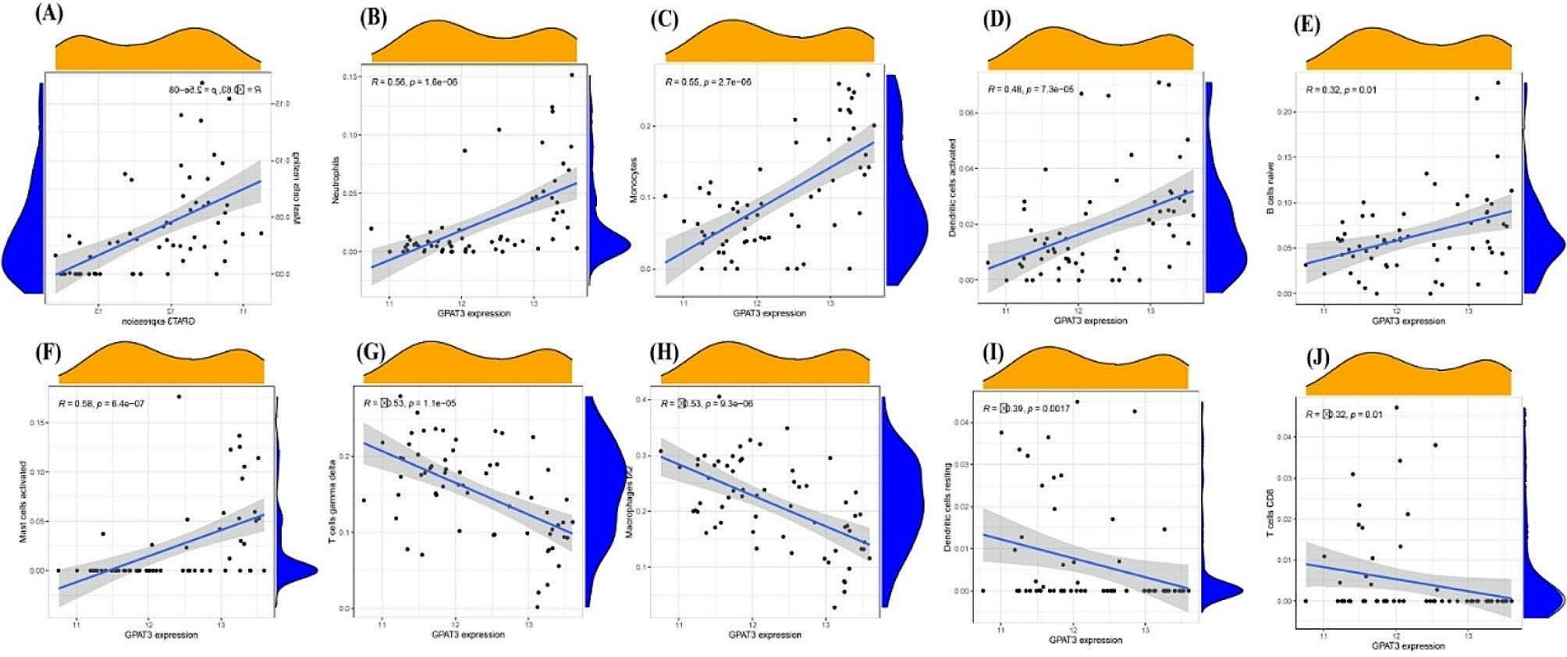
Correlation between GPAT3 and infiltrating immune cells in NAFLD and healthy samples. (A. Mast cells resting), (B. Neutrophils), (C. Monocytes), (D. Dendritic cells activated), (E. B cells naive), (F. Mast cells activated), (G. T cells gamma delta), (H. Macrophages M2), (I. Dendritic cells resting), (J. T cells CD8)

**Fig. 7:**
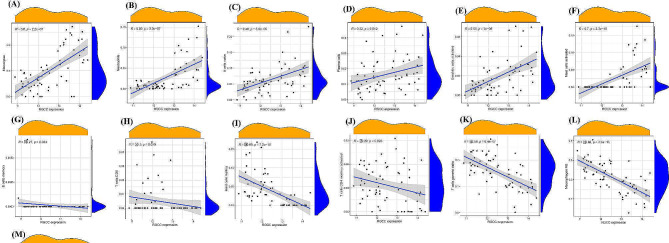
Correlation between RGCC and infiltrating immune cells in NAFLD and healthy samples. (A. Monocytes), (B. Neutrophils), (C. B cells naive), (D. Plasma cells), (E. Dendritic cells activated), (F. Mast cells activated), (G. B cells memory), (H. T cells CD8), (I. Mast cells resting), (J. T cells CD4 memory activated), (K. T cells gamma delta), (L. Macrophages M2), (M. Dendritic cells resting)

**Fig. 8:**
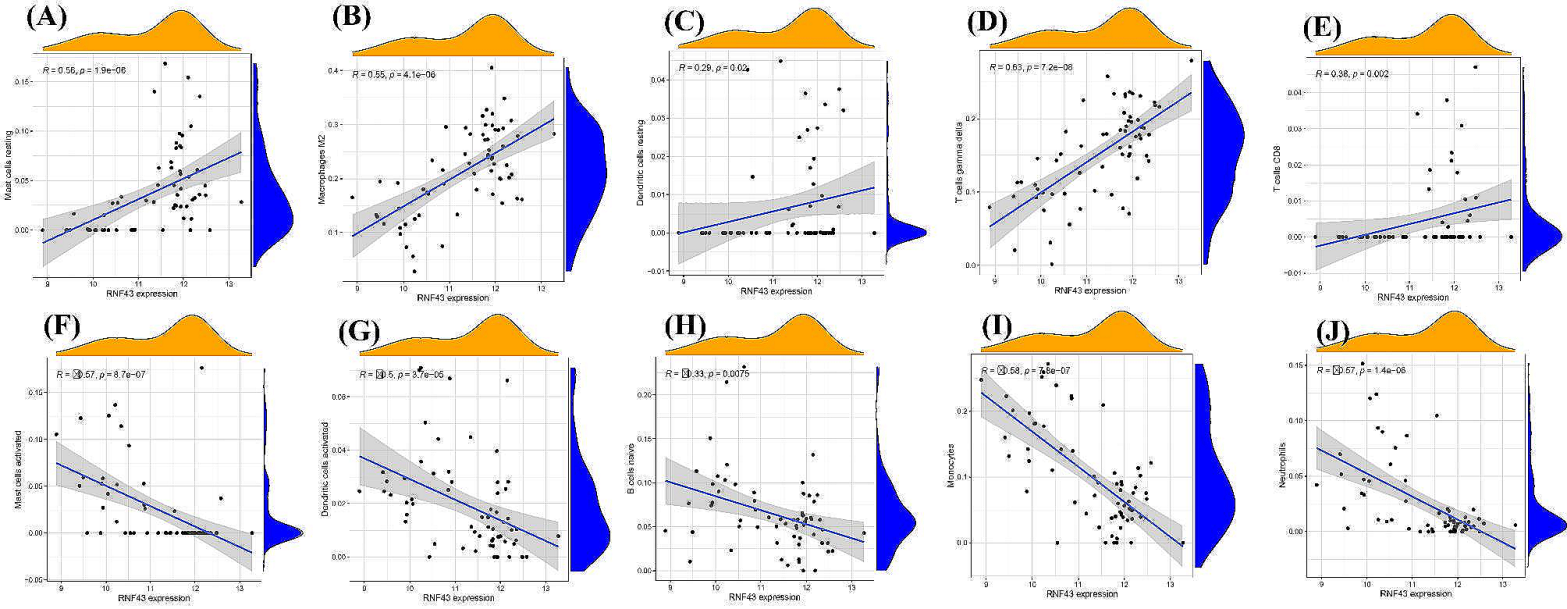
Correlation between RNF43 and infiltrating immune cells in NAFLD and normal samples. (A. Mast cells resting), (B. Macrophages M2), (C. Dendritic cells resting), (D. T cells gamma delta), (E. T cells CD8), (F. Mast cells activated), (G. Dendritic cells activated), (H. B cells naive), (I. Monocytes), (J. Neutrophils)

Next, the relationships between the expression levels of *FOSB*, *GPAT3*, *RGCC*, and *RNF43* and the infiltration levels of immune cells were explored. *FOSB* was positively correlated with activated mast cells, monocytes, neutrophils (Fig. 5), (A. Mast cells resting), (B. T cells gamma delta), (C. Macrophages M2), (D. T cells CD4 memory activated), (E. Dendritic cells resting), (F. T cells CD8), (G. Dendritic cells activated), (H. B cells naive), (I. Monocytes), (J. T cells follicular helper), (K. Neutrophils), (L. Mast cells activated). Meanwhile, *GPAT3* was positively correlated with activated mast cells, neutrophils (Fig. 6), (A. Mast cells resting), (B. Neutrophils), (C. Monocytes), (D. Dendritic cells activated), (E. B cells naive), (F. Mast cells activated), (G. T cells gamma delta), (H. Macrophages M2), (I. Dendritic cells resting), (J. T cells CD8). Moreover, *RGCC* was positively correlated with activated mast cells, neutrophils, monocytes, activated dendritic cells, naive B cells, plasma cells, while negatively associated with memory B cells, resting dendritic cells (Fig. 7), (A. Monocytes), (B. Neutrophils), (C. B cells naive), (D. Plasma cells), (E. Dendritic cells activated), (F. Mast cells activated), (G. B cells memory), (H. T cells CD8), (I. Mast cells resting), (J. T cells CD4 memory activated), (K. T cells gamma delta), (L. Macrophages M2), (M. Dendritic cells resting). Finally, *RNF43* was positively correlated with γδ T cells, M2 macrophages (Fig. 8), (A. Mast cells resting), (B. Macrophages M2), (C. Dendritic cells resting), (D. T cells gamma delta), (E. T cells CD8), (F. Mast cells activated), (G. Dendritic cells activated), (H. B cells naive), (I. Monocytes), (J. Neutrophils). These data suggest that *FOSB*, *GPAT3*, *RGCC*, and *RNF43* may be involved in the progression of NAFLD by modulating some types of immune cells.

### Validation of the expression of four diagnostic genes

**Fig. 9:**
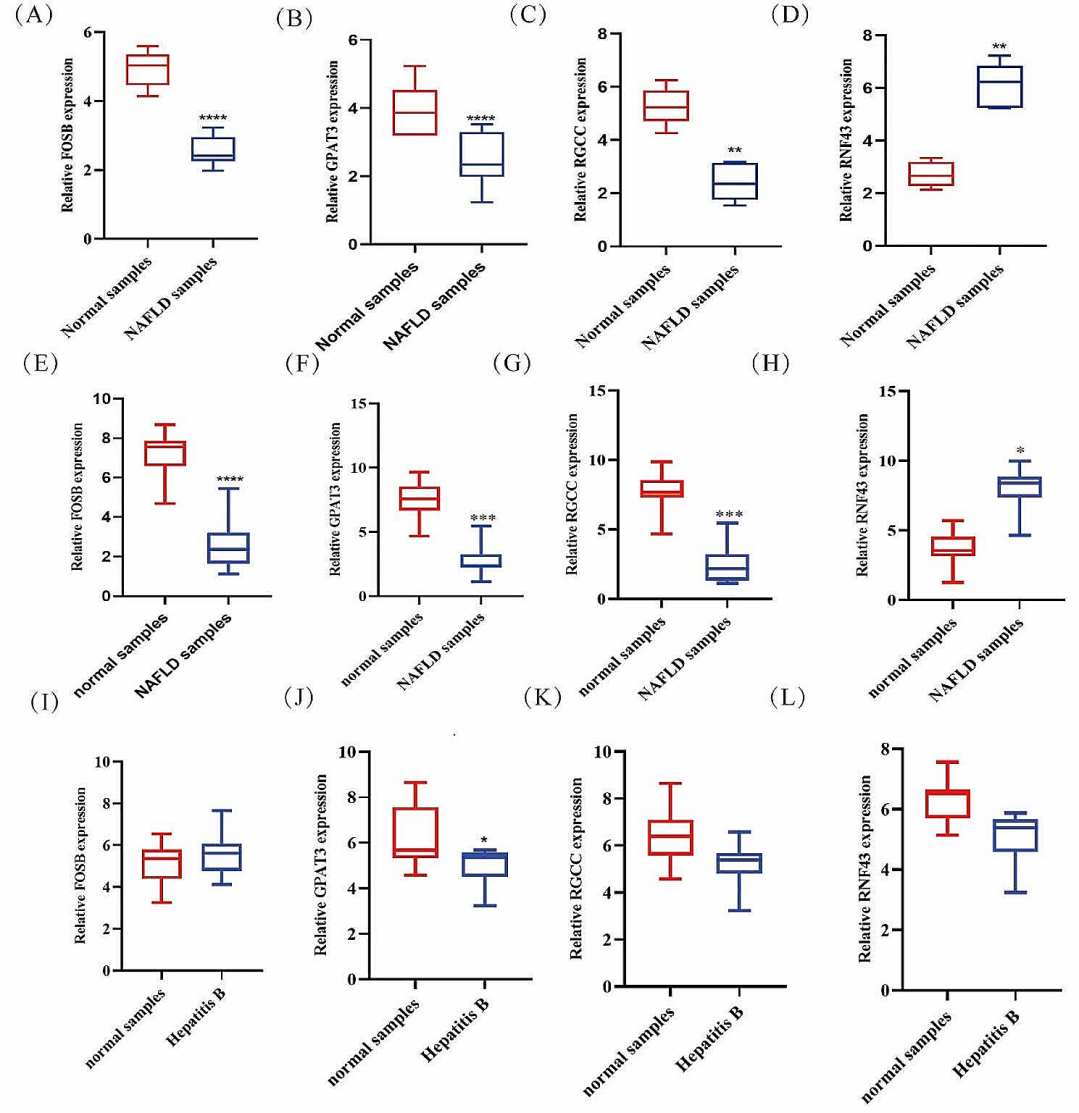
mRNA levels of FOSB (**A**), GPAT3 (**B**), RGCC (**C**) and RNF43 (**D**) in NAFLD mice samples and normal control determined by RT-PCR. mRNA levels of FOSB (**E**-**H**), GPAT3 (**I**-**L**) in NAFLD human samples and normal samples from our cohort determined by RT-PCR

RT-PCR was used to validate the expression of *FOSB*, *GPAT3*, *RGCC*, and *RNF43* in the livers of NAFLD model mice. Compared with the normal mice, the expression levels of *FOSB*, *GPAT3*, and *RGCC* were significantly reduced (Fig. 9A, B and C), while the expression level of *RNF43* was significantly increased (Fig. 9D) in the NAFLD mice. We further confirmed the mRNA expression of these four genes in the blood of NAFLD patients (Fig. 9E-H). The data showed similar changes as in mice. These results further suggest that *FOSB*, *GPAT3*, *RGCC*, and *RNF43* may be potential diagnostic biomarkers of NAFLD.

**Fig. 10:**
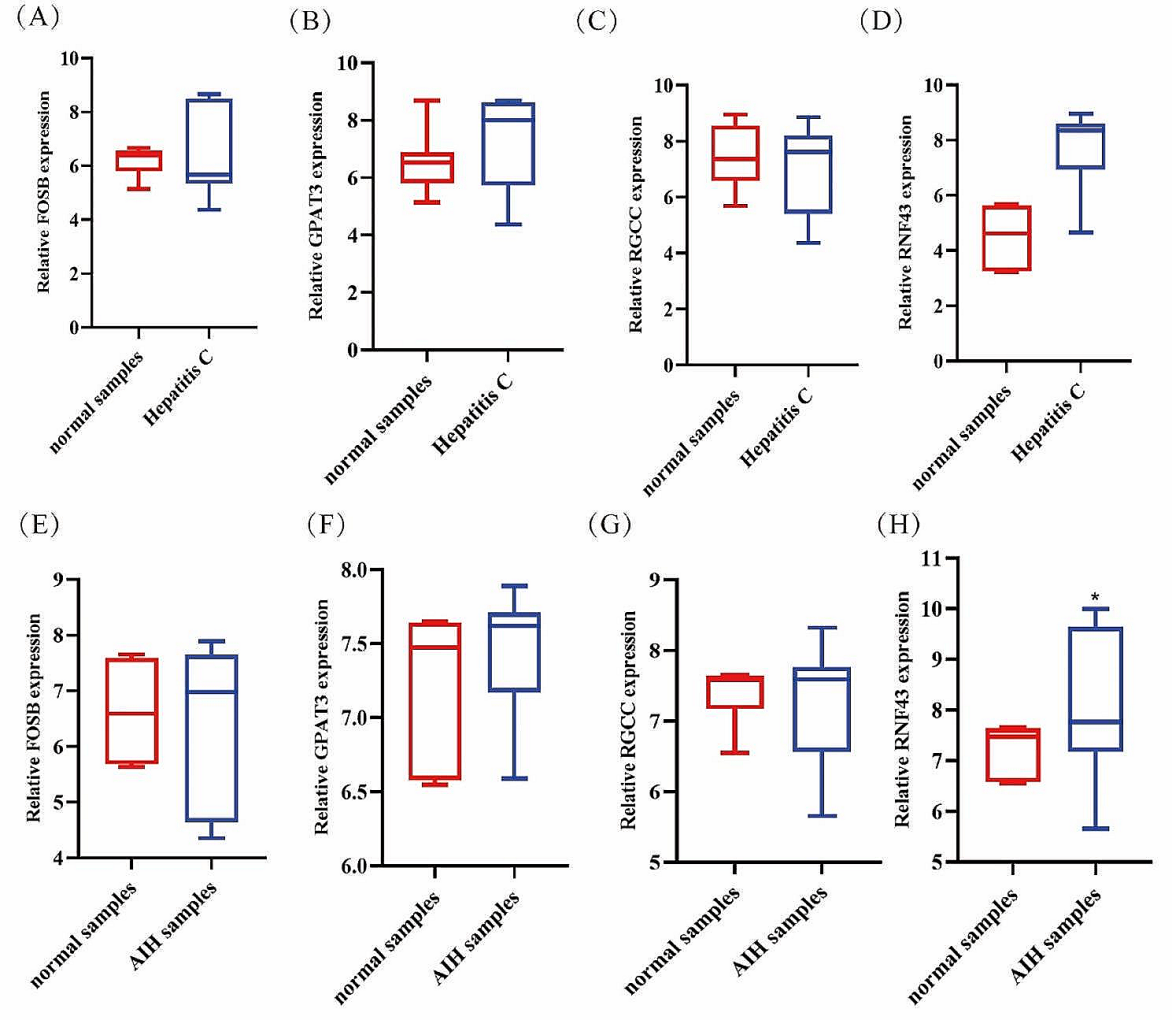
mRNA levels of RGCC (**A**-**D**) and RNF43 (**E**-**H**) in NAFLD human samples and normal samples from our cohort determined by RT-PCR

We also detected the expression of *FOSB*, *GPAT3*, *RGCC*, and *RNF43* in the blood of patients with hepatitis B (Fig. 9I-L), hepatitis C (Fig. 10A-D) and AIH (Fig. 9E-F). Compared with the healthy people, the expression levels of *GPAT3* was significantly reduced (Fig. 9J), while the expression level of *FOSB*, *RGCC*, and *RNF43* showed no significant change (Fig. 9I, K, L) in patients with hepatitis B; the expression levels of *RNF43* was significantly increased (Fig. 10D), while the expression level of *FOSB*, *RGCC*, and *GPAT3* showed no significant change (Fig. 9A, B, C) in patients with hepatitis C; the expression level of *RGCC* was significantly reduced (Fig. 9G), while the expression level of *RNF43* was significant increased (Fig. 9H) in the AIH patients. These results further suggest that combine *FOSB*, *GPAT3*, *RGCC*, and *RNF43* may be potential diagnostic biomarkers of NAFLD.

## Discussion

NAFLD is the most common type of liver disease that affects many individuals worldwide [27]. Hepatitis, cirrhosis and liver cancer caused by NAFLD are global public health problems [3, 28–31]. The prevalence of NAFLD in our population is increasing annually, and the proportion of new cases of NAFLD is about 4% each year [32]. There is a significant difference in the prevalence rate of NAFLD in different regions, with the prevalence rate in the economically developed eastern and southern regions being higher than that in the central and western inland regions. The difference in lifestyles could also an important reason for the difference in prevalence rates [33]. Over the past two decades, the burden of NAFLD has increased significantly with China’s booming economy and radical lifestyle changes. The early diagnosis of NAFLD is essential for the treatment of this disease, Unfortunately, there are no diagnostic tools that can allow a prompt diagnosis [34, 35].

In our study, machine learning methods were used to find DEGs. We analyzed the GEO dataset and identified 334 DEGs between NAFLD and healthy samples. GO analysis showed that these 334 DEGs were mainly involved in chemical reactions, negative regulation of cell processes, organic material and cell-to-chemical stimulation reactions. KEGG pathway analysis showed that cytokine–cytokine receptor interactions, the interleukin (IL)-17 signaling pathway, the tumor necrosis factor (TNF) signaling pathway, and transcriptional dysregulation were obviously enriched. IL-17 is an inflammatory factor induced by Th17 cells, which can stimulate endothelial cells and epithelial cells to produce other inflammatory factors and chemokines, resulting in tissue damage. Overnutrition mediates DNA damage in hepatocytes through nonclassical prefoldin RPB5 interaction factor. DNA damage induces inflammation and neutrophilic infiltration of white adipose tissue through Th17 and IL-17 A, which in turn mediates insulin resistance and fatty acid release. Fatty acids are stored in the liver as triglycerides, leading to the development of NAFLD and NASH. TNF works through two receptors TNFR1 (also known as p55) and TNFR2 (also known as p75). TNFR1 is expressed in most tissues, and interacts with TNF, leading to a classic pro-inflammatory response, mostly through activation of the NF-κB and c-Jun pathways. Studies have that a high-fat diet increases the expression of TNF-a in the body, which further promotes the activation of NF-κB and TLRs signaling pathway, leading to the increase of inflammatory factors such as IL-6, and ultimately the formation of chronic inflammation. These results suggest that the selected DEGs are actively involved in the inflammatory process and are essential for the progression of NAFLD [36].

To identify potential diagnostic genes for NAFLD, two machine learning algorithms were used to analyze the 334 DEGs, thus four genes (*FOSB*, *GPAT3*, *RGCC*, and *RNF43*) were identified. FOSB is an early hepatocyte response protein that is essential for lipid metabolism [37]. Studies have shown that the expression of FOSB is significantly downregulated in patients with fatty liver disease [38]. Meanwhile, GPAT3 is the first critical rate-limiting enzyme in the glycerol-3-phosphate synthesis pathway [39]. Therefore, regulating the synthesis of triglycerides in the liver and reducing the accumulation of excess lipids in hepatocytes may be used as a novel therapeutic strategy to improve hepatic steatosis, and preventing and treating NAFLD [40]. Moreover, *RGCC*, also known as C13, is an important complement response gene that is widely expressed in various tissues [41]. It can regulate cell cycle, promote cell proliferation and differentiation [42], and regulate the immune response [43]. Interestingly, deletion or mutation of *RNF43* was found to lead to lipid accumulation and liver inflammation in non-obese mice fed a with normal diet. In humans, mutation of *RNF43* increases the risk of liver diseases such as fatty liver disease and liver cancer, as well as reduces the life expectancy of patients [44]. In addition, the ROC method confirmed their strong ability to differentiate NAFLD samples from normal samples. Thus, our findings suggest that *FOSB*, *GPAT3*, *RGCC*, and *RNF43* are potential diagnostic biomarkers for NAFLD.

We also examined *FOSB*, *GPAT3*, *RGCC*, and *RNF43* expression in patients with hepatitis B, hepatitis C, and AIH. There are some consistencies between the mouse liver transcriptome and the human blood transcriptome [45]. To compensate for the difficulty of obtaining human liver samples mouse liver could be used to detect the expression of some genes [46]. The results showed an increased expression of *FOSB* in patients with hepatitis B, an increased expression of *RNF43* in patients with hepatitis C, and an increased expression of *RNF43* along with a decreased expression of *RGCC* in patients with AIH. These results were consistent with the expression profile in patients with NAFLD. Many studies have suggested that *FOSB*, *RNF43* and *RGCC* are closely related to the inflammatory response of immune cells [47, 48]. Hepatitis B virus and hepatitis C virus infection can stimulate the inflammatory response of activated liver immune cells, thus change the expression of these genes will. However, hepatitis B, hepatitis C and AIH can only be diagnosed by the detection of corresponding virus genes and antibodies. In addition, FOSB is reported to be related to hepatitis C [49], while RNF43 and GPAT3 are related to hepatitis B [50, 51]. Our results show that in the diagnosis of NAFLD, the ROC value of the four gene expressions in blood is close to 1. It is suggested that the simultaneous changes of four genes are closely related to NAFLD. Therefore, combination of these four gene expressions in blood might be an alternative diagnostic method to the gold diagnostic standard (liver biopsy) with high sensitivity, specificity and safety.

Liver biopsy is the current gold standard for the diagnosis of NAFLD. Although it can directly observe the pathological features of the liver to diagnose NAFLD, it is inconvenient for patients to tolerate side effects such as pain and discomfort after puncture due to the trauma. Additionally, the technique of liver puncture biopsy is complicated, and many clinicians are not skilled enough resulting in dirty biopsy. However, for safety and financial cost, ultrasound which is the primary screening tool in clinical practice, is being used to diagnose NAFLD. Recently, a meta-analysis showed that the sensitivity of diagnosing moderate and severe fatty liver with ultrasound was 84.8% and the specificity was 93.6%. However, ultrasound has its drawbacks, its diagnostic sensitivity is only 55% when the fat content of the liver is below 20%. Although ultrasound screening for NAFLD is safer than liver tissue biopsy, it is not diagnostic of early NAFLD due to the clinician’s experience. Our results show that the sensitivity of mRNA analysis using blood can reach a maximum of 0.985, thus reducing damage to the body. We compared the expression of the genes in patients with hepatitis B, C, and autoimmune hepatitis and found that these genes were specifically expressed in patients with nonalcoholic fatty liver. Compared to biopsy, our diagnostic method is highly safe, which requires only a small amount of fingertip blood irrespective of the physician’s skill. It is more acceptable to the patients as it is less harmful to the body. Compared to imaging, the diagnosis by finger blood is not influenced by the physician’s experience and is more sensitive and specific for NAFLD.

In recent years, multiple studies have indicated that immune cell infiltration plays a crucial role in the occurrence and development of NAFLD. The progression of NAFLD is closely related to macrophages (Kupffer cells) [52], neutrophils [53], dendritic cells [54], and natural killer (NK) T cells [55]. Kupffer cells constitute about 20% of nonparenchymal hepatocytes and are involved in T lymphocyte activation and tolerance. Furthermore, TNF-α is mainly derived from Kupffer cells. In response to high-fat diet stimulation, the TNF-α levels are significantly increased in Kupffer cells, and large amounts of proinflammatory factors such as IL-6 are released to aggravate the inflammatory response [56]; additionally, stimuli such as a high-fat diet also increase the number of Kupffer cells themselves, thereby changing the activation state of NK T cells and inducing cell necrosis and apoptosis [57]. Moreover, increased oxidative stress activates Kupffer cells and the corresponding macrophages, and increases the levels of chemokines and cytokines [58]; thus, it plays a pivotal role in the progression of NAFLD. Therefore, it is necessary to evaluate the infiltration of immune cells and to identify the diversity of infiltrating immune cell components to reveal the molecular mechanisms in NAFLD to find new immunotherapeutic targets.

In this study, we observed dysregulated levels of CD8^+^ T cells, resting dendritic cells, γδ T cells, M2 macrophages, resting mast cells, neutrophils, and naive B cells between normal and NAFLD samples. Moreover, the histamine produced during the degranulation of mast cells in the liver of patients with NAFLD induces an inflammatory response that damages the surrounding tissues. In livers from NAFLD mice, mast cells promote the progression of NAFLD to NASH hepatitis by upregulating the aldehydrogenase 1 family member A3 and downregulating microRNA-144-3. In contrast, mast-cell deficient mice exhibit improved the symptoms of NAFLD. The change of M1/M2 polarization may induce hepatic inflammation and hepatocyte injury, thus activating satellite cells. After activation, satellite cells release large numbers of collagen fibers, which promote liver fibrosis. In addition, neutrophils [59], CD4^+^ T cells [60], CD8^+^ T cells [61], NK T cells [62], T helper 17 cells [63], and other immune cells have been confirmed to participate in the development of NAFLD. In the liver, these different types of cells regulate each other through the expression and release of inflammatory cytokines, chemokines, interleukins, and other cytokines in autocrine and paracrine modes, thus maintaining liver homeostasis. The abnormal expression of these secreted factors can cause necrosis and apoptosis of hepatocytes, activate satellite cells, and promote the development of NAFLD/NASH. Our data indicate that FOSB, GPAT3, RGCC, and RNF43 may participate in the progression of NAFLD by modulating some immune cells. Nevertheless, further studies are warranted to confirm these findings.

## Conclusion

In this study, bioinformatics analysis indicates that *FOSB*, *GPAT3*, *RGCC*, and *RNF43* are the key DEGs for the comparison of NAFLD with healthy specimens. The results of this study provide novel insights into the potential molecular causality of NAFLD and potential therapeutic targets for NAFLD. Therefore, ongoing research focuses in refining the diagnostic value of existing biomarkers as well as discovering novel markers in the areas of epigenetics, transcriptomics and metabolomics, in the prospect of early reliable diagnosis that would guide targeted care.

### Electronic supplementary material

Below is the link to the electronic supplementary material.

Supplementary Material 1

Supplementary Material 2

## Data Availability

All data are available in the main text or the supplementary materials.

## Abbreviations

NAFLD: non-alcoholic fatty liver disease
GO: gene oncology
KEGG: Kyoto Encyclopedia of Genes and Genomes
LDL: low-density lipoprotein cholesterol
hPSC: human pluripotent stem cells
HCC: hepatocellular carcinoma
FGF: fibroblast growth factor
FGF21: Fibroblast growth factor 21
