# Supplementary material for: An novel effective and safe model for the diagnosis of nonalcoholic fatty liver disease in China: gene excavations, clinical validations, and mechanism elucidation
Source: J Transl Med. 2024 Jul 4;22:624. doi: 10.1186/s12967-024-05315-3 (PMC11225259; doi:10.1186/s12967-024-05315-3)
Supplement: Supplementary file 1 — Supplementary Material 1 [file 12967_2024_5315_MOESM1_ESM.docx]

**Replies to Reviewer 1**

1. The first few results can be combined as the authors simply mention their gene findings and not clearly explaining the impact of their findings.

**Response:** Thank you for your helpful recommendation. We have combined the first few results to explain the impact of the results of our study. (page 2, lines 2–25; page 3, lines 1–3).

***Abstract***

***Background:*** *Non-alcoholic fatty liver disease (NAFLD) is one of the most common chronic liver diseases. NAFLD leads to liver fibrosis and hepatocellular carcinoma, and it also has systemic effects associated with metabolic diseases, cardiovascular diseases, chronic kidney disease, and malignant tumors. Therefore, it is important to diagnose NAFLD early to prevent these adverse effects.*

***Methods:*** *The GSE89632 dataset was downloaded from the Gene Expression Omnibus database, and then the optimal genes were screened from the data cohort using lasso and Support Vector Machine Recursive Feature Elimination (SVM-RFE). The ROC values of the optimal genes for the diagnosis of NAFLD were calculated. The relationship between optimal genes and immune cells was determined using the DECONVOLUTION algorithm CIBERSORT. Finally, the specificity and sensitivity of the diagnostic genes were verified by detecting the expression of the diagnostic genes in blood samples from 320 NAFLD patients and liver samples from 12 mice.*

***Results:****Through machine learning we identified FOSB, GPAT3, RGCC and RNF43 were the key diagnostic genes for NAFLD, and they were further demonstrated by a receiver operating characteristic curve analysis. We found that the combined diagnosis of the four genes identified NAFLD samples well from normal samples (AUC = 0.997). FOSB, GPAT3, RGCC and RNF43 were strongly associated with immune cell infiltration. We also experimentally examined the expression of these genes in NAFLD patients and NAFLD mice, and the results showed that these genes are highly specific and sensitive.*

***Conclusions:*** *Data from both clinical and animal studies demonstrate the high sensitivity, specificity and safety of FOSB, GPAT3, RGCC and RNF43 for the diagnosis of NAFLD. The relationship between diagnostic key genes and immune cell infiltration may help to understand the development of NAFLD. The study was reviewed and approved by Ethics Committee of Tianjin Second People's Hospital in 2021 (ChiCTR1900024415).*

2. Not enough context is offered for the non bio informatic reasecher about the reason for moving from one gene finding metholody to another. Starting the result sections with overarching explanations will help create the flow better.

**Response:** We appreciate the reviewer's feedback. To address this concern, we have combined the result sections and added explanations to improve the flow (page 9, lines 18–19; page 9-10, lines 25–1; page 10, lines 12; page 10, lines 23–24; page 11, lines 6–7; Page 11, lines 16–19;).

Page 9, lines 18–19: ***Identification of DEGs in NAFLD***

*Firstly, we find the differentially up-regulated or down-regulated genes in the NAFLD samples in the dataset.*

Page 9-10, lines 25–1: ***Functional Enrichment Analyses***

*Next, GO and KEGG analyses were performed for these 334 DEGs using R software with the Cluster Profile package.*

Page 10, lines 12: ***Identification of Diagnostic Marker Candidates***

*Two different algorithms were used to identify potential biomarkers.*

Page 10, lines 23–24: *Compared with the healthy samples, the expression levels of FOSB, GPAT3, and RGCC were significantly downregulated in the NAFLD samples (****Fig. 2D, E, F****); whereas the expression level of RNF43 was significantly upregulated in the NAFLD samples (****Fig. 2G****).*

Page 11, lines 6–7: *Then, ROC curve was used to analyze the diagnostic value of combined two, three and four genes for NAFLD.*

Page 11, lines 16–19: *Immune cell infiltration in the tumor microenvironment is an independent predictor of overall survival and prognosis. Therefore, we investigated the coefficients of FOSB, GPAT3, RGCC, and RNF43 and the infiltration status of immune cells in NAFLD and normal samples to determine the correlation between them.*

Page 12, lines 11–12: *RT-PCR was used to validate the expression of FOSB, GPAT3, RGCC, and RNF43 in the livers of NAFLD model mice.*

3. There should be a concluding statement at the end of each result for the reader to string the story as they go.

**Response:** Thank you for your suggestion. We have added concluding statements at the end of each result to aid the reader in understanding the narrative. (page 9, lines 22–23; page 10, lines 7–10; page 10, lines 19-20; page 11, lines 2–5; page 11, lines 11–17; Page 12, lines 7–9; Page 12, lines 15–18).

Page 9, lines 22–23: *We identified 334 DEGs, including 223 upregulated genes and 111 downregulated genes (Fig. 1 A).*

Page 10, lines 7–10: *KEGG analysis showed that the following pathways were significantly enriched: cytokine–cytokine receptor interactions, the interleukin (IL)-17 signaling pathway, the tumor necrosis factor (TNF) signaling pathway, and transcriptional dysregulation in cancer (Fig. 1 E).*

Page 10, lines 19-20: *These four genes (FOSB, GPAT3, RNF43, and RGCC) may be the key genes involved in the progression of NAFLD.*

Page 11, lines 2–5: *We found that the AUCs of FOSB (****Fig. 2H****), GPAT3 (****Fig. 2I****), RGCC (****Fig. 2J****), and RNF43 (****Fig. 2K****) were 0.974, 0.983, 0.985, and 0.958, respectively, suggesting that all four genes showed a strong ability to differentiate NAFLD samples from normal samples.*

Page 11, lines 11–17: *Each gene alone was used to diagnose NAFLD at a very high level, and the combination of all four genes had the highest ROC value for the diagnosis of NAFLD.*

Page 12, lines 7–9: *These data suggest that FOSB, GPAT3, RGCC, and RNF43 may be involved in the progression of NAFLD by modulating some types of immune cells.*

Page 12, lines 15–18: *We further confirmed the mRNA expression of these four genes in the blood of NAFLD patients (****Fig. 9 E-H****). The data showed similar changes as in mice. These results further suggest that FOSB, GPAT3, RGCC, and RNF43 may be potential diagnostic biomarkers of NAFLD.*

**Replies to Reviewer 2**

1. A correction should be addressed by the authors as follows: The abstract is not well organized; the sentences are incomplete, and there is no sense of continuity. It would be feasible if you included the significance of the current study in the abstract.

**Response:** We are extremely grateful to the reviewer for highlighting this issue. We have rewritten the abstract to improve its organization and coherence while discussing the significance of our study. (page 2, lines 2–25; page 3, lines 1–3).

Page 2, lines 2–25; page 3, lines 1–3: ***Abstract***

***Background:*** *Non-alcoholic fatty liver disease (NAFLD) is one of the most common chronic liver diseases. NAFLD leads to liver fibrosis and hepatocellular carcinoma, and it also has systemic effects associated with metabolic diseases, cardiovascular diseases, chronic kidney disease, and malignant tumors. Therefore, it is important to diagnose NAFLD early to prevent these adverse effects.*

***Methods:*** *The GSE89632 dataset was downloaded from the Gene Expression Omnibus database, and then the optimal genes were screened from the data cohort using lasso and Support Vector Machine Recursive Feature Elimination (SVM-RFE). The ROC values of the optimal genes for the diagnosis of NAFLD were calculated. The relationship between optimal genes and immune cells was determined using the DECONVOLUTION algorithm CIBERSORT. Finally, the specificity and sensitivity of the diagnostic genes were verified by detecting the expression of the diagnostic genes in blood samples from 320 NAFLD patients and liver samples from 12 mice.*

***Results:****Through machine learning we identified FOSB, GPAT3, RGCC and RNF43 were the key diagnostic genes for NAFLD, and they were further demonstrated by a receiver operating characteristic curve analysis. We found that the combined diagnosis of the four genes identified NAFLD samples well from normal samples (AUC = 0.997). FOSB, GPAT3, RGCC and RNF43 were strongly associated with immune cell infiltration. We also experimentally examined the expression of these genes in NAFLD patients and NAFLD mice, and the results showed that these genes are highly specific and sensitive.*

***Conclusions:*** *Data from both clinical and animal studies demonstrate the high sensitivity, specificity and safety of FOSB, GPAT3, RGCC and RNF43 for the diagnosis of NAFLD. The relationship between diagnostic key genes and immune cell infiltration may help to understand the development of NAFLD. The study was reviewed and approved by Ethics Committee of Tianjin Second People's Hospital in 2021 (ChiCTR1900024415).*

2. A brief description of how the authors selected information from the literature in the databases, as well as what time period they searched for, is missing.

**Response:** Thank you for your helpful recommendation. We have briefly described the process of information selection from the literature and mentioned the corresponding time period in the Microarray Data section. (page 6, lines 10–18).

Page 6, lines 10–18: *The microarray data used to establish the diagnostic model for NAFLD were the GSE89632 mRNA expression profile data (including blood samples from 19 NAFLD patients and 24 normal controls), was downloaded from GEO (*[*https://www.ncbi.nlm.nih.gov/geo/*](https://www.ncbi.nlm.nih.gov/geo/)*) [25]. Screening criteria: the period of data collection was before January 2023. The dataset intelligently contained healthy people and patients with NAFLD (excluding patients with alcoholic fatty liver disease, hepatitis, liver fibrosis, and hepatocellular carcinoma). The patients with fatty liver disease had not been subjected to pharmacological interventions as at the time of data collection.*

3. The authors should justify and expand the information on the advantages of this work for biomedical applications.

**Response:** We appreciate your suggestion. Accordingly, we have added a discussion on the advantages of this study for biomedical applications in the discussion section. (page 13, lines 6–17; page 16, lines 4–23)

Page 13, lines 6–17: *NAFLD is the most common type of liver disease that affects many individuals worldwide [27]. Hepatitis, cirrhosis and liver cancer caused by NAFLD are global public health problems [3, 28-31]. The prevalence of NAFLD in our population is increasing annually, and the proportion of new cases of NAFLD is about 4% each year [32]. There is a significant difference in the prevalence rate of NAFLD in different regions, with the prevalence rate in the economically developed eastern and southern regions being higher than that in the central and western inland regions. The difference in lifestyles could also an important reason for the difference in prevalence rates [33]. Over the past two decades, the burden of NAFLD has increased significantly with China's booming economy and radical lifestyle changes. The early diagnosis of NAFLD is essential for the treatment of this disease, Unfortunately, there are no diagnostic tools that can allow a prompt diagnosis [34, 35]. Two machine learning algorithms, lasso and support vector machine recursive feature elimination (SVM-RFE), were used to predict the NAFLD status using possible diagnostic factors. Lasso and support vector machine recursive feature elimination (SVM-RFE) represent classical methodologies utilized for screening candidate diagnostic genes within datasets. Presently, liver biopsy stands as the benchmark for NAFLD diagnosis, emphasizing the need for alternative diagnostic modalities with high sensitivity and specificity. Four diagnostic genes have been identified and experimentally validated using machine learning frameworks demonstrating high safety along with elevated levels of sensitivity and specificity, which position these genes as potential candidates for NAFLD diagnosis.*

4. Authors should specify the main experimental conditions used based on the evidence from the literature. Where they briefly describe the most important data reported in the literature in a homogeneous manner and reinforce the relevance of this method as novel alternatives. Authors should discuss whether the use of work represents a solid alternative to existing therapeutics. The article lacks some background information on the significance of studying. Providing a brief introduction or literature review on the potential sources would enhance the relevance of the study. I recommend that the authors cite and comment on other previous studies that evaluated the effect of this work. There are excellent studies that should be mentioned in the introduction.

**Response:** Thanks for your insightful comments. We have addressed the missing background information by providing a brief introduction and literature review on the significance of the study. Additionally, we have cited and commented on previous studies recommended by the reviewer in the introduction (page 16, lines 4–23).

Page 16, lines 4–23: *Liver biopsy is the current gold standard for the diagnosis of NAFLD. Although it can directly observe the pathological features of the liver to diagnose NAFLD, it is inconvenient for patients to tolerate side effects such as pain and discomfort after puncture due to the trauma. Additionally, the technique of liver puncture biopsy is complicated, and many clinicians are not skilled enough resulting in dirty biopsy. However, for safety and financial cost, ultrasound which is the primary screening tool in clinical practice, is being used to diagnose NAFLD. Recently, a meta-analysis showed that the sensitivity of diagnosing moderate and severe fatty liver with ultrasound was 84.8% and the specificity was 93.6%. However, ultrasound has its drawbacks, its diagnostic sensitivity is only 55% when the fat content of the liver is below 20%. Although ultrasound screening for NAFLD is safer than liver tissue biopsy, it is not diagnostic of early NAFLD due to the clinician's experience. Our results show that the sensitivity of mRNA analysis using blood can reach a maximum of 0.985, thus reducing damage to the body. We compared the expression of the genes in patients with hepatitis B, C, and autoimmune hepatitis and found that these genes were specifically expressed in patients with nonalcoholic fatty liver. Compared to biopsy, our diagnostic method is highly safe, which requires only a small amount of fingertip blood irrespective of the physician's skill. It is more acceptable to the patients as it is less harmful to the body. Compared to imaging, the diagnosis by finger blood is not influenced by the physician’s experience and is more sensitive and specific for NAFLD.*

**REFERENCES** **13**: *Eftekhari, Aziz, et al. "Hepatoprotective role of berberine against paraquat-induced liver toxicity in rat." Environmental Science and Pollution Research 27 (2020): 4969-4975.*

**REFERENCES** **15**: *Baran, Ayşe, et al. "Ecofriendly synthesis of silver nanoparticles using ananas comosus fruit peels: anticancer and antimicrobial activities." Bioinorganic Chemistry and Applications 2021 (2021): 1-8.*

**REFERENCES** **18**: *Gunashova, G. Y. "Synthesis of silver nanoparticles using a thermophilic bacterium strain isolated from the spring Yukhari istisu of the Kalbajar region (Azerbaijan)." Advances in Biology and Earth Sciences 7.3 (2022): 198-204.*

**REFERENCES** **17**: *Baran, Ayşe, et al. "Investigation of antimicrobial and cytotoxic properties and specification of silver nanoparticles (AgNPs) derived from Cicer arietinum L. green leaf extract." Frontiers in Bioengineering and Biotechnology 10 (2022): 855136.*

**Replies to Reviewer 3**

1. It is recommended that the formatting of the body content be carefully revised to be consistent, either by indenting the first line by two spaces or by aligning the two paragraphs.

**Response:** Thank you for your insightful suggestion. We have carefully revised the formatting of the body content to ensure consistent formatting.

2. The descriptions of Figures 4B, 4C and 4D are not found in the main text.

**Response:** Thank you for bringing this to our attention. We have updated the main text to include descriptions of Figures 4B, 4C, and 4D.

3. It is recommended that the images in Figure 6 be reworked for clarity and grouped appropriately.

**Response:** We appreciate your suggestion. To enhance clarity, we have reworked the images in Figure 6 by increasing their pixels and regrouping them appropriately.

4. Figure 7-Figure supplement 10 Why is the figure note below the image Supplementary Figure 7-Supplementary Figure 10?

**Response:** Thank you for highlighting this inconsistency. We have made the necessary adjustments to ensure that the figure notes align correctly with Supplementary Figures 7-10.

5. Some of the fonts in Figure 11 are obscured, and it is recommended that they be re-grouped appropriately.

**Response:** Thank you for your insightful suggestion. We have re-grouped the fonts in Figure 11 for better visibility.

6. It is recommended that the following references related to methodologies be cited in appropriate statements in the Materials and methods to enrich the article. PMID: 37476183, PMID：38027604.

**Response:** We thank you for your recommendations regarding the references. The suggested references have been cited in relevant statements within the Materials and Methods section, and they have also been added to the reference list.

**REFERENCES** **25**: *Gao P, Wang H, Li H, Shu L, Han Z, Li S, Cheng H, Dai X: miR-21-5p Inhibits the Proliferation, Migration, and Invasion of Glioma by Targeting S100A10. J Cancer 2023, 14:1781-1793.*

**REFERENCES** **26**: *Li H, Dai X, Zhou L, Nie J, Cheng H, Gao P: Ferroptosis-related gene MTF-1 as a novel prognostic biomarker in low-grade glioma and its correlation with immune infiltration. Heliyon 2023, 9:e21159.*

7. Please carefully check the format of some references in the manuscript and modify them in strict accordance with the requirements of this publication.

**Response:** We appreciate your attention to detail. We have carefully checked the format of references in the manuscript and modified them in strict accordance with the requirements of the publication.

8. The language and grammar of this manuscript need to be further polished.

**Response:** Thank you for your observation. We acknowledge the need for further refinement of the language and grammar. Therefore, our manuscript has been revised by native English-speaking foreign teachers and international students.
