# Supplementary material for: An novel effective and safe model for the diagnosis of nonalcoholic fatty liver disease in China: gene excavations, clinical validations, and mechanism elucidation
Source: J Transl Med. 2024 Jul 4;22:624. doi: 10.1186/s12967-024-05315-3 (PMC11225259; doi:10.1186/s12967-024-05315-3)
Supplement: Supplementary file 2 — Supplementary Material 2 [file 12967_2024_5315_MOESM2_ESM.doc]

Re: Manuscript Number: JTRM-D-23-10364 “*An novel effective and safe model for the diagnosis of nonalcoholic fatty liver disease in China: gene excavations, clinical validations, and mechanism elucidation”*

Dear Editor,

Thank you very much for your email regarding the revision of our manuscript “**An novel effective and safe model for the diagnosis of nonalcoholic fatty liver disease in China: gene excavations, clinical validations, and mechanism elucidation**” (Manuscript Number: JTRM-D-23-10364).

We greatly thank you and the reviewers for your valuable comments. These comments are of great significance to our research. We have revised our manuscript according to your and the reviewer s’ comments, and the amendments are highlighted in yellow in the revised version of our manuscript attached as “Manuscript.docx”. The revised parts are marked with line numbers, and we have also uploaded a clean version of our revised manuscript as “Manuscript (clean).docx”. Moreover, according to the reviewers’ suggestions, we have also improved our manuscript in several parts, and a point-by-point response to the reviewers’ comments was uploaded as “JTRM-D-23-10364-Response.docx”.

We believe that the revised manuscript is very much improved by the revisions. We appreciate you and the reviewers’ great help and efforts on this manuscript. We kindly hope the revised manuscript is acceptable for publication in *Journal of Translational Medicine*.

Yours sincerely

Yaogang Wang, PhD (Corresponding author)

Professor

School of Public Health

Tianjin Medical University

Qixiangtai Road 22, Heping District, 300070, Tianjin, China

Tel: +86 22 5959 6228; Email: YaogangWANG@tmu.edu.cn
